# Identification and Inference with Invalid Instruments

**DOI:** 10.1146/annurev-statistics-112723-034721

**Published:** 2024-09-25

**Authors:** Hyunseung Kang, Zijian Guo, Zhonghua Liu, Dylan Small

**Affiliations:** 1 Department of Statistics, University of Wisconsin–Madison, Madison, Wisconsin, USA; 2 Department of Statistics, Rutgers University, Piscataway, New Jersey, USA; 3 Department of Biostatistics, Columbia University, New York, NY, USA; 4 Department of Statistics and Data Science, The Wharton School, University of Pennsylvania, Philadelphia, Pennsylvania, USA

**Keywords:** heteroskedasticity, instrumental variables, invalid instruments, Mendelian randomization

## Abstract

Instrumental variables (IVs) are widely used to study the causal effect of an exposure on an outcome in the presence of unmeasured confounding. IVs require an instrument, a variable that (a) is associated with the exposure, (b) has no direct effect on the outcome except through the exposure, and (c) is not related to unmeasured confounders. Unfortunately, finding variables that satisfy conditions b or c can be challenging in practice. This article reviews works where instruments may not satisfy conditions b or c, which we refer to as invalid instruments. We review identification and inference under different violations of b or c, specifically under linear models, nonlinear models, and heteroskedastic models. We conclude with an empirical comparison of various methods by reanalyzing the effect of body mass index on systolic blood pressure from the UK Biobank.

## 1. INTRODUCTION

Instrumental variables (IVs) are a popular tool to estimate the causal effect of an exposure on an outcome in the presence of unmeasured confounders, which are unmeasured variables that affect both the exposure and the outcome. Briefly, IVs require finding a variable called an instrument that satisfies three core conditions: (a) the instrument is related to the exposure, (b) the instrument has no direct pathway to the outcome except through the exposure, and (c) the instrument is not related to unmeasured confounders that affect the exposure and the outcome. Details are provided by Hernán & Robins (2006) and Baiocchi et al. (2014) and in Section 2.1. Condition b is often referred to as the exclusion restriction (Imbens & Angrist 1994, Angrist et al. 1996). IVs can be a valuable tool in settings where a randomized trial, which is the gold standard for dealing with unmeasured confounders, is impractical.

There is often uncertainty about whether candidate instruments do, in fact, satisfy conditions a-c, especially conditions b and c; this problem is loosely referred to as the invalid instruments problem (Murray 2006, Conley et al. 2012). For example, if the instrument is a genetic marker, which is the case in Mendelian randomization (MR) (Davey Smith & Ebrahim 2003, 2004), satisfying condition b would imply that the genetic marker’s only biological function is to affect the exposure. However, this assumption is untenable for many genetic markers as they often have multiple biological functions, a phenomenon broadly known as pleiotropy (Solovieff et al. 2013, Hemani et al. 2018). More generally, without a complete understanding of the instruments’ downstream effects on the outcome, IVs are plagued by possible violations of condition b. Also, when condition a is weakly satisfied in that the instrument’s association to the exposure is small, also referred to as weak instruments (Staiger & Stock 1997, Stock et al. 2002, Andrews et al. 2019), a slight violation of condition b can lead to dramatically biased estimates of the causal effect of the exposure (Bound et al. 1995, Hahn & Hausman 2005, Small & Rosenbaum 2008).

Recently, some promising frameworks have been developed to study the identification and inference of the causal effect of the exposure when some candidate instruments are, in fact, invalid. In this article, we organize these frameworks into the following categories:

1. **Linear models:** One of the earliest works in the IV literature generalizes a popular linear, IV model to parametrize the violations of conditions b and c. Roughly speaking, instruments are invalid under linear models if they have nonzero, partial effects on the outcome after conditioning on the exposure. Some key contributions in this area include those of Han (2008), Kolesár et al. (2015), Kang et al. (2016b), Guo et al. (2018), Windmeijer et al. (2019, 2021), Fan & Wu (2024), and Guo (2023). Given that the invalid instruments violate conditions b and c in a linear fashion, these authors present simple conditions for identification, most notably the majority rule. The majority rule states that a majority of the instruments are valid, without knowing which instruments are valid a priori. Also, some of these works have relatively straightforward methods for estimation, such as the median estimator. However, if the linear outcome model is mis-specified, these methods can lead to wrong conclusions about the effect of the exposure.
2. **Nonlinear models:** Recent work in this area utilizes the nonlinearities in the exposure model when instruments do not satisfy conditions b and c. For example, Guo et al. (2022) proposed finding nonlinear trends in the exposure model via machine learning methods to identify and infer the causal effect of the exposure. Sun et al. (2023) proposed using higher-order interactions of instruments and G-estimation (Robins et al. 1992, Robins 1994) to identify and infer the causal effect of the exposure. Critically, both of these methods rely on the exposure model’s nonlinearity for identification, and it is important to check the required nonlinearity conditions to ensure that the identification conditions are plausible with the data (Lewbel 2019).
3. **Heteroskedastic models:** These works utilize heteroskedsaticity in the exposure model or the outcome model to identify the causal effect with invalid instruments. Some prominent studies in this area include those of Lewbel (2012), Tchetgen Tchetgen et al. (2021), Sun et al. (2022), Ye et al. (2024), and Liu et al. (2023). Similar to the nonlinear modeling framework above, these methods require heteroskedasticity, and the required heteroskedasticity condition should be checked in practice.

The rest of the article goes into the details behind each category above. We also empirically compare the methods discussed in the article by reanalyzing the causal effect of body mass index (BMI) on blood pressure from the UK Biobank. We do not discuss in detail an important interplay between weak instruments and invalid instruments and the challenges that they pose. The intersection between weak instruments and invalid instruments has recently gained considerable attention, and we provide a summary of the recent work in this area plus other remaining questions in the study of invalid instruments at the end of the article.

## 2. LINEAR MODELS

### 2.1. Model and Definition of Invalid Instruments

Let Y∈R denote the outcome, D∈R denote the exposure, and $Z\in R^{p}$ denote the p instruments. Consider the following model for Y,D, and Z where, without loss of generality, we assume that Y,D, and Z are centered to mean zero:

1. $$\begin{array}{rrr} D=Z^{⊤}\gamma +\delta , & \; & \text{E}(\delta |Z)=0, \end{array}$$

2. $$\begin{array}{ccc} Y=D\beta +Z^{⊤}\pi +\epsilon , & \; & \text{E}(\epsilon |Z)=0. \end{array}$$

In Equation 1, the parameter $\gamma \in R^{p\times 1}$ represents the instruments’ relevance to the exposure. For this review, we only consider the case where all instruments are relevant (i.e., $\gamma_{j}\neq 0$ for all j=1,…,p) in order to focus on invalid instruments. For extensions to identifying and inferring the causal effect when some instruments are not relevant and some instruments are invalid, readers are directed to Guo et al. (2018) and Fan & Wu (2024).

In Equation 2, the parameter β∈R represents the effect of the exposure on the outcome and is the main parameter of interest in IVs. The parameter β is sometimes referred to as a structural parameter (Goldberger 1972, Wooldridge 2010, Angrist & Pischke 2009) to distinguish it from the usual regression coefficient or a reduced-form parameter, which can often be estimated consistently with ordinary least squares (OLS). Specifically, the estimate of the regression coefficients from running an ordinary least squares regression of Y on D and Z, denoted as ${\overset{ˆ}{\beta }}_{\text{OLS}}$, is generally inconsistent for β because the variable D is not independent of the error term ϵ. In contrast, for the parameters in Equation 1, we can run a ordinary least squares regression of D on Z to obtain a consistent estimator of γ.

The parameter $\pi \in R^{p\times 1}$ represents the effects of the instruments on the outcome after adjusting for the exposure. If π=0, Equation 2 reduces to a well-studied, linear IV model (Angrist & Pischke 2009, Wooldridge 2010) and all p instruments are said to be valid instruments. If π≠0, some of the p instruments are not valid and the nonzero elements of π encode which of the instruments are invalid.

**Definition 1 (valid instrument).** Suppose Equations 1 and 2 hold. Instrument j∈{1,…,p} is valid if $\pi_{j}=0$ and invalid if $\pi_{j}\neq 0$. Let $𝒱=\left\{j:\pi_{j}=0\right\}$ be the set of valid instruments.

If the set of valid instruments 𝒱 is known a priori and there is at least one valid instrument (i.e., the size of the set, denoted as |𝒱|, is greater than or equal to 1), Equation 2 again reduces to a well-studied linear IV model where the complement of 𝒱 serves as the control variables (for an example, see Wooldridge 2010, chapter 5). But, in practice, the knowledge about 𝒱 is unknown, and the central goal under the invalid IV framework is to study identification and inference of the causal effect when there is no a priori knowledge about which instruments among the p candidate instruments are valid.

Definition 1 of a valid instrument is closely related to the definition of a valid instrument under an additive, linear, constant effects (ALICE) potential outcomes model (Holland 1988). Let Y(d,z)∈R be the potential outcome if an individual were to have exposure d∈R and instruments $z\in R^{p}$. For $d,d^{\prime }\in R$ and $z,z^{\prime }\in R^{p}$, the ALICE model states

3. $$Y\left(d^{\prime },z^{\prime }\right)-Y(d,z)=\left(d^{\prime }-d\right)\tilde{\beta }+{\left(z^{\prime }-z\right)}^{⊤}\tilde{\psi },\;\text{E}[Y(0,0)|Z]=Z^{⊤}\tilde{\phi }.$$

The tilde notation in Equation 3 emphasizes that the model parameters are from the potential outcomes model. The parameter $\tilde{\beta }$ represents the causal effect of changing the exposure by one unit on the outcome. Each jth element of $\tilde{\psi }\in R^{p\times 1}$ represents the causal effect of changing the jth instrument by one unit on the outcome. The term $\text{E}[Y(0,0)|Z]=Z^{⊤}\tilde{\phi }$, where $\tilde{\phi }\in R^{p\times 1}$, represents the effect from unmeasured confounding. If the no direct effect condition b is formalized as $Y(d,z)=Y\left(d,z^{\prime }\right)$ for all $d,z,z^{\prime }$, we have $\tilde{\psi }=0$. If the no instrument confounding condition c is formalized as Z⊥Y(d,z), where ⊥ stands for independence between two random variables, we have $Z^{⊤}\tilde{\phi }=0$. Also, if we assume the stable unit treatment value assumption (Rubin 1980) or causal consistency where Y=Y(D,Z), Equation 3 simplifies to Equation 2 where $\tilde{\beta }=\beta ,\pi =\tilde{\psi }+\tilde{\phi }$, and ϵ=Y(0,0)-E[Y(0,0)∣Z]. In other words, under the ALICE model for potential outcomes and causal consistency, the violations of conditions b and c are summarized with the parameter π.

We now make some other important remarks about the definition of a valid instrument. First, the validity of instrument j depends on the candidate set of instruments. Instrument j may be valid with one set of instruments but may not be valid with another set of instruments. Second, if we have covariates X that are independent of the error terms in Equations 1 and 2, we can adjust for X by first fitting a linear regression of Y on X,D on Z, and Z on X. Then we replace Y,D, and Z in Equations 1 and 2 with the residuals of the linear regressions from the first step. This procedure is justified by the Frisch–Waugh–Lovell theorem (Davidson & MacKinnon 1993). Third, several studies (Hahn & Hausman 2005; Berkowitz et al. 2008, 2012; Conley et al. 2012; Guggenberger 2012; Armstrong & Kolesár 2021) have studied the properties of existing estimators and tests of β when there is a near violation of instrument validity. In its simplest form, a near violation of instrument validity is characterized as $\tilde{\psi }=0,\tilde{\phi }=C/\sqrt{n}$ or as $\pi =C/\sqrt{n}$ for some constant vector $C\in R^{p}$, and n is the sample size. These authors showed that existing estimators will be biased and tests of β will have an inflated type I error rate. Fourth, Liao (2013), Cheng & Liao (2015), DiTraglia (2016), and Patel et al. (2024) considered estimating β when there is a known set of valid instruments and another set of potentially invalid instruments. Finally, we remark that there are methods to construct bounds of β (Small 2007, Baiocchi et al. 2010, Ashley & Parmeter 2015, Kang et al. 2016a, Fogarty et al. 2021) under various assumptions about the magnitude of π.

### 2.2. Identification of the Causal Effect of the Exposure with Invalid Instruments

To better understand how β can be identified when the set of valid instruments 𝒱 is unknown, it is useful to consider a model of Y that only depends on Z, often referred to as a reduced-form model:

4. $$Y=Z^{⊤}\Gamma +e,\;\;\Gamma =\beta \gamma +\pi ,e=\beta \delta +\epsilon ,\text{E}(e|Z)=0.$$

With the observed data Y,D, and Z, we can identify the parameters $\gamma \in R^{p\times 1}$ and $\Gamma \in R^{p\times 1}$ based on the following relationships from ordinary least squares:

$$\Gamma =\text{E}{\left(ZZ^{⊤}\right)}^{-1}\text{E}\left(ZY\right)\;\text{and}\;\gamma =\text{E}{\left(ZZ^{⊤}\right)}^{-1}\text{E}\left(ZD\right).$$

With the parameters Γ and γ identified from the data, we can reframe identifying β as finding a unique value of (β,π) from (γ,Γ) based on the system of linear equations in Equation 4 (i.e., Γ=βγ+π). Also, the system of linear equations reveals the role that 𝒱 plays in identifying β. For example, suppose π=0 so that all instruments are valid and 𝒱={1,…,p}. Then, the linear system of equations simplifies to Γ=βγ and we can identify β given Γ and γ by setting $\beta =\Gamma_{j}/\gamma_{j}$ for any j=1,…,p. Or, suppose the set of valid instruments 𝒱 is known a priori and there is at least one valid instrument (i.e., |𝒱|≥1). Then, for j∈𝒱, the system of linear equations simplifies to $\Gamma_{j}=\beta \gamma_{j}$ and we can again identify β. Finally, if there are no restrictions on π, there are no unique values of (β,π) given (γ,Γ) since there are p linear equations and p+1 unknown variables.

When 𝒱 is unknown, Han (2008) and Kang et al. (2016b) proposed what is now called the majority rule to identify β:

5. $$\left|𝒱\right|>\frac{p}{2}.$$

Simply put, the majority rule states that the number of valid instruments is more than 50% of the instruments. Critically, we do not have to know a priori which instruments are valid; we simply have to know that the majority of the instruments are valid.

We briefly illustrate how the majority rule in Equation 5 places a constraint on the linear system of equations above to identify β. The full proof is provided by Kang et al. (2016b), who prove a necessary and sufficient condition to have a unique solution of (β,π) given (γ,Γ) and a lower bound on |𝒱|. Given (γ,Γ), let (β,π) and $\left(\beta^{\prime },\pi^{\prime }\right)$ be the solutions to the system of equations, i.e., Γ=βγ+π and $\Gamma =\beta^{\prime }\gamma +\pi^{\prime }$. Let 𝒱 and ${𝒱}^{\prime }$ denote the sets of valid instruments defined by π and $\pi^{\prime }$, respectively. By the majority rule, $𝒱\cap {𝒱}^{\prime }\neq \emptyset$ and we can always pick $j\in 𝒱\cap {𝒱}^{\prime }$ where $\pi_{j}=\pi_{j}^{\prime }=0$. For instrument j, the system of linear equations simplifies to $\Gamma_{j}=\beta \gamma_{j}$ and $\Gamma_{j}=\beta^{\prime }\gamma_{j}$, which imply that $\beta =\beta^{\prime }$. Furthermore, we have $\pi =\Gamma -\beta \gamma =\Gamma -\beta^{\prime }\gamma =\pi^{\prime }$, and thus, the solution to the system of linear equations is unique.

We can also construct a falsification test of the majority rule where the null hypothesis assumes that the majority rule holds. For example, suppose the majority rule holds, and the nonzero elements of π are far away from 0. In this setting, there should only be one large cluster of instruments with the same $\Gamma_{j}/\gamma_{j}$, and the size of this cluster should be greater than p/2. If we do not see such a cluster, this indicates a violation of the majority rule. For additional details on how to conduct this test, readers are directed to Guo et al. (2023).

Next, Guo et al. (2018) proposed the plurality rule to identify β:

6. $$\left|𝒱\right|>\underset{c\neq 0}{\text{max}}\left|{ℐ}_{c}\right|\;\text{with}\;{ℐ}_{c}=\left\{j\in \left\{1,\ldots ,p\right\}:\frac{\pi_{j}}{\gamma_{j}}=c\right\}.$$

In words, ${ℐ}_{c}$ denotes a subset of instruments that have a common value of $\Gamma_{j}/\gamma_{j}$, specifically $\Gamma_{j}/\gamma_{j}=\beta +c$. Note that the set of valid instruments 𝒱 equals to ${ℐ}_{0}$ where c=0. The plurality condition requires that the set of valid instruments 𝒱 is the largest among all subsets of instruments with a common value of $\Gamma_{j}/\gamma_{j}$ that is not equal to β. Also, if the majority rule holds, the plurality rule holds since the size of the set ${ℐ}_{c}$ for any c≠0 cannot be greater than p/2, i.e., $p/2>{\text{max}}_{c\neq 0}\left|{ℐ}_{c}\right|$. In other words, the majority rule is a sufficient condition for the plurality rule. Finally, we can also falsify the plurality rule, albeit under more stringent conditions (for details, see Guo et al. 2023).

We conclude with a remark about the work by Andrews (1999), who primarily focused on developing a model selection procedure to consistently estimate 𝒱. The model selection procedure relies on using a test statistic called the J test (Hansen 1982) to distinguish between valid instruments and invalid instruments, and a couple of the methods discussed below use this procedure to tune relevant tuning parameters. Also, when characterizing the properties of the proposed model selection procedure, Andrews (1999) proposed a condition for identifying β that is a version of the plurality rule. Specifically, for any subset 𝒞⊆{1,…,p} and a vector $v\in R^{p}$, let $v_{𝒞}\in R^{\left|𝒞\right|}$ be the vector with elements defined by the subset 𝒞. Then, Andrews (1999) stated that β is identified if

$$\forall 𝒞\;\text{where}\;\left|𝒞\right|\geq \left|𝒱\right|\;\text{and}\;𝒱\neq 𝒞,\;\pi_{𝒞}\neq q\gamma_{𝒞},$$

for any q≠0.

### 2.3. Estimation and Inference of the Causal Effect of the Exposure

The next two sections describe estimation and inference for the causal effect of the exposure. The first section discusses consistent estimation of the exposure effect. The second section discusses constructing confidence intervals for the exposure effect, starting with pointwise inference and concluding with uniform inference.

#### 2.3.1. Consistent estimators of β.

We lay out the following notation to describe different estimators of β. For each study unit i=1,…,n, let $\left(Y_{i},D_{i},Z_{i}\right)\in R⊗R⊗R^{p}$ be the observed outcome, exposure, and p instruments, respectively. Let $Y=\left(Y_{1},\ldots ,Y_{n}\right)\in R^{n\times 1},D=\left(D_{1},\ldots ,D_{n}\right)\in R^{n\times 1}$, and $Z=\left(Z_{1},\ldots ,Z_{n}\right)\in R^{n\times p}$. As before, without loss of generality, we assume that the vectors Y and D as well as the columns of the matrix Z are centered to mean zero. Let $P_{Z}=Z{\left(Z^{⊤}Z\right)}^{-1}Z^{⊤}\in R^{n\times n}$ be the projection matrix onto the column space of Z and let $P_{Z}^{⊥}=I-P_{Z}\in R^{n\times n}$ be the residual projection matrix where $I\in R^{n\times n}$ is the identity matrix. For any vector $v\in R^{p}$ and q≥1, let $\|v{\|}_{q}={\left(\sum_{j=1}^{p}v_{j}^{q}\right)}^{1/q}$ be its q norm and let $v_{j}$ denote the jth element of v. Finally, for a set 𝒱⊆{1,…,p}, let ${𝒱}^{c}$ denote its complement and $Z_{𝒱}\in R^{n\times |𝒱|}$ denote the matrix Z with the columns specified by 𝒱.

We start by describing the two-stage least squares (TSLS) estimator, a popular estimator of β when 𝒱 is known a priori and |𝒱|≥1. The TSLS estimator first fits a linear regression model of D on Z and obtains the predicted values of D, i.e., $P_{Z}D$. Second, it fits a linear regression model of Y on $P_{Z}D$ and $Z_{{𝒱}^{c}}$. If ${𝒱}^{c}$ is an empty set (i.e., all of the instruments are valid), we drop the $Z_{{𝒱}^{c}}$ term in the second regression model. The TSLS estimator of β is the estimated regression coefficient in front of $P_{Z}D$ from the second regression model. More succinctly, given a set of valid IVs 𝒱 and |𝒱|≥1, the TSLS is the solution to the following optimization problem:

7. $$\left[{\overset{ˆ}{\beta }}_{\text{TSLS}}(𝒱),{\overset{ˆ}{\pi }}_{\text{TSLS}}(𝒱)\right]={\text{argmin}}_{\beta ,\pi_{{𝒱}^{c}}}\frac{1}{2}{\left\|P_{Z}\left(Y-D\beta -Z_{{𝒱}^{c}}\pi_{{𝒱}^{c}}\right)\right\|}_{2}^{2}.$$

Some methods for invalid instruments compare their proposed estimators of β, which do not know 𝒱 a priori, with the TSLS estimator with a known 𝒱; if used in this context, the TSLS estimator is sometimes referred to as the oracle estimator (Guo et al. 2018, Windmeijer et al. 2021). If their proposed estimator of β is asymptotically equivalent to the oracle estimator, the proposed estimator is said to be oracle-optimal in the literature.

One of the first methods to estimate β when 𝒱 is unknown a priori is the median estimator of Han (2008). Consider the ordinary least squares estimators of Γ and γ:

$$\overset{ˆ}{\Gamma }={\left(Z^{⊤}Z\right)}^{-1}Z^{⊤}Y,\;\overset{ˆ}{\gamma }={\left(Z^{⊤}Z\right)}^{-1}Z^{⊤}D.$$

Under mild assumptions (see Wooldridge 2010, chapter 4), $\overset{ˆ}{\Gamma }$ and $\overset{ˆ}{\gamma }$ are asymptotically normal:

8. nΓˆγˆ-Γγ→dN00,ΩΓΩΓγΩΓγ⊤Ωγ.

We remark that the covariance matrices $\Omega_{\Gamma }\in R^{p\times p},\Omega_{\Gamma \gamma }\in R^{p\times p}$, and $\Omega_{\gamma }\in R^{p\times p}$ can be consistently estimated. The median estimator of β, denoted as ${\overset{ˆ}{\beta }}_{\text{med}}$, can be written as the median of p ratios of ${\hat{\Gamma }}_{j}/{\overset{ˆ}{\gamma }}_{j},j=1,\ldots ,p$:

9. $${\overset{ˆ}{\beta }}_{\text{med}}=\text{median}\left\{{\tilde{\beta }}_{1},\ldots ,{\tilde{\beta }}_{p}\right\},\;{\tilde{\beta }}_{j}=\frac{{\hat{\Gamma }}_{j}}{{\overset{ˆ}{\gamma }}_{j}}.$$

Han (2008) established that ${\overset{ˆ}{\beta }}_{\text{med}}$ is consistent if the majority rule in Equation 5 holds. Later, Windmeijer et al. (2019) established that the limiting distribution of the median estimator is an order statistic of a normal distribution. However, inference based on the median estimator is challenging due to the nonnegligible bias of the order statistic (Windmeijer et al. 2019, Guo et al. 2023).

Kang et al. (2016b) proposed a lasso-based estimator of β, which the authors referred to as sisVIVE (some invalid, some valid IV estimator). sisVIVE is inspired by the TSLS estimator in Equation 7 and directly solves for the model parameters in Equation 2 with a penalty term on π:

10. $$\left[{\overset{ˆ}{\beta }}_{\text{sisVIVE}},{\overset{ˆ}{\pi }}_{\text{sisVIVE}}\right]=\underset{\beta ,\pi }{\text{argmin}}\frac{1}{2}{\left\|P_{Z}(Y-D\beta -Z\pi )\right\|}_{2}^{2}+\lambda \|\pi {\|}_{1},\;\lambda >0.$$

This optimization problem can be solved with existing penalized regression software by reformulating Equation 10 as follows:

$${\overset{ˆ}{\pi }}_{\text{sisVIVE}}=\underset{\pi }{\text{argmin}}\frac{1}{2}{\left\|\left(P_{Z}-P_{P_{Z}D}\right)(Y-Z\pi )\right\|}_{2}^{2}+\lambda \|\pi {\|}_{1},$$

$${\overset{ˆ}{\beta }}_{\text{sisVIVE}}=\frac{{\left(P_{Z}D\right)}^{⊤}\left(Y-Z{\overset{ˆ}{\pi }}_{\text{sisVIVE}}\right)}{{\left\|P_{Z}D\right\|}_{2}^{2}}.$$

The first step of the two-step procedure estimates π by using existing software for the lasso (e.g., Efron et al. 2004). The second step is a dot product between two n-dimensional vectors. Kang et al. (2016b) established conditions when ${\overset{ˆ}{\beta }}_{\text{sisVIVE}}$ is consistent for β and recommended choosing the tuning parameter λ by cross-validation. Later, Bao et al. (2019) studied the finite sample properties of ${\overset{ˆ}{\beta }}_{\text{sisVIVE}}$ through a simulation study.

Finally, Kolesár et al. (2015) showed that the k-class estimator from Anatolyev (2013), denoted as ${\overset{ˆ}{\beta }}_{\text{kclass}}$ and formalized as

$${\overset{ˆ}{\beta }}_{\text{kclass}}=\frac{D^{⊤}\left(I-kP_{Z}^{⊥}\right)Y}{D^{⊤}\left(I-kP_{Z}^{⊥}\right)D}\;\text{where}\;k=\frac{1-\frac{1}{n}}{1-\frac{p}{n}-\frac{1}{n}},$$

is consistent for β when both the number of instruments p and the number of samples n grow to infinity and the following orthogonality condition between π and γ hold:

11. $$\frac{1}{n}\pi^{⊤}Z^{⊤}Z\gamma \to 0\;\text{where}\;\frac{p}{n}\to c\in \left[0,1\right).$$

In words, the orthogonality condition states that the effect of the instruments on the exposure (i.e., γ) is orthogonal to the direct effect of the instruments on the outcome (i.e., π). If all the instruments are mutually independent of each other, the orthogonality condition roughly translates to $\pi^{⊤}\gamma \approx 0$. Under this case, the system of linear equations in Equation 4 can be rewritten as $\Gamma^{⊤}\gamma \approx \beta \gamma^{⊤}\gamma$ and the parameter β can be identified given (γ,Γ).

Unfortunately, beyond consistency, ${\overset{ˆ}{\beta }}_{\text{med}}$ and ${\overset{ˆ}{\beta }}_{\text{sisVIVE}}$ have no inferential guarantees, such as having a limiting normal distribution to enable testing $H_{0}:\beta =0$ or constructing a confidence interval for β. Also, to establish asymptotic normality of ${\overset{ˆ}{\beta }}_{\text{kclass}}$, Kolesár et al. (2015) further assumed that the parameters γ and π are random and follow a multivariate normal distribution. The next two sections discuss some progress on conducting inference about β.

#### 2.3.2. Pointwise inference of β.

We start with Windmeijer et al. (2019), who proposed to use an adaptive version of the sisVIVE estimator. Specifically, consider the adaptive lasso (Zou 2006) version of the sisVIVE estimator in Equation 10 where the initial estimator is the median estimator in Equation 9:

$${\overset{ˆ}{\pi }}_{\text{med}}=\overset{ˆ}{\Gamma }-\overset{ˆ}{\gamma }{\overset{ˆ}{\beta }}_{\text{med}},$$

$${\overset{ˆ}{\pi }}_{\text{AdLasso}}=\underset{\pi }{\text{argmin}}\frac{1}{2}{\left\|\left(P_{Z}-P_{P_{Z}D}\right)(Y-Z\pi )\right\|}_{2}^{2}+\lambda \sum_{j=1}^{p}\frac{1}{\left|{\overset{ˆ}{\pi }}_{\text{med},j}\right|}\left|\pi_{j}\right|,\;\lambda >0,$$

$${\overset{ˆ}{\beta }}_{\text{AdLasso}}=\frac{{\left(P_{Z}D\right)}^{⊤}\left(Y-Z{\overset{ˆ}{\pi }}_{\text{adp}}\right)}{{\left\|P_{Z}D\right\|}_{2}^{2}}.$$

Windmeijer et al. (2019) showed that ${\overset{ˆ}{\beta }}_{\text{AdLasso}}$ is consistent and asymptotically normal if the majority rule holds. They also proposed a method to choose λ by using a downward testing procedure of Andrews (1999).

Guo et al. (2018) proposed a different approach, called two-stage hard thresholding (TSHT), to conduct inference on β. Broadly speaking, TSHT treats each instrument j as a voter and uses a plurality voting procedure to estimate β. Specifically, consider a voting matrix $H\in R^{p\times p}$ where $H_{j,k}=1$ if the pair of instruments j and k yield similar estimates of β and $H_{j,k}=0$ if the pair yield different estimates of β, i.e.,

12. $$H_{j,k}=\left\{\begin{array}{ll} 1 & \text{if}\;\left|\frac{{\hat{\Gamma }}_{j}}{{\overset{ˆ}{\gamma }}_{j}}-\frac{{\hat{\Gamma }}_{k}}{{\overset{ˆ}{\gamma }}_{k}}\right|\leq z_{1-\frac{\alpha }{2p}}\cdot \hat{\text{se}}\left(\frac{{\hat{\Gamma }}_{j}}{{\overset{ˆ}{\gamma }}_{j}}-\frac{{\hat{\Gamma }}_{k}}{{\overset{ˆ}{\gamma }}_{k}}\right),\\ 0 & \text{otherwise.} \end{array}\right.$$

The term α∈(0,1) is the prespecified significance level, $z_{1-\alpha /2p}$ is the 1-α/2p quantile of the standard normal distribution, and $\hat{\text{se}}\left({\hat{\Gamma }}_{j}/{\overset{ˆ}{\gamma }}_{j}-{\hat{\Gamma }}_{k}/{\overset{ˆ}{\gamma }}_{k}\right)$ is the estimated standard error of the difference between ${\hat{\Gamma }}_{j}/{\overset{ˆ}{\gamma }}_{j}$ and ${\hat{\Gamma }}_{k}/{\overset{ˆ}{\gamma }}_{k}$. For each j=1,…,p, let ${\left\|H_{j,\cdot }\right\|}_{0}=\sum_{k=1}^{p}H_{j,k}$ denote the number of nonzero elements in the jth row of H (i.e., $H_{j,\cdot }$). The norm ${\left\|H_{j,\cdot }\right\|}_{0}$ measures the number of instruments that are close to the jth instrument’s ratio ${\hat{\Gamma }}_{j}/{\overset{ˆ}{\gamma }}_{j}.\;{\left\|H_{j,\cdot }\right\|}_{0}$ can also be thought of as the number of votes that instrument j received on being the valid instrument. From ${\left\|H_{j,\cdot }\right\|}_{0}$, we can estimate the set of valid instruments by picking instruments that received a majority or a plurality of votes:

$${\overset{ˆ}{𝒱}}_{\text{TSHT}}≔\left\{j\in \{1,\ldots ,p\}\left|{\left\|H_{j,\cdot }\right\|}_{0}>\frac{p}{2}\right.\right\}\cup \left\{j\in \{1,\ldots ,p\}|{\left\|H_{j,\cdot }\right\|}_{0}=\underset{k}{\text{max}}\;{\left\|H_{k,\cdot }\right\|}_{0}\right\}.$$

After estimating 𝒱, we can use the TSLS estimator with the set ${\overset{ˆ}{𝒱}}_{\text{TSHT}}$ to estimate β. Under the plurality rule, Guo et al. (2018) showed that this estimator, denoted as ${\overset{ˆ}{\beta }}_{\text{TSHT}}$, is consistent, asymptotically normal, and oracle-optimal for β. Zhang et al. (2022) recently proposed an improvement of TSHT that prevents choosing a large number of irrelevant instruments through a resampling method. They showed that their procedure is effective in regimes where the number of instruments p is very large.

Windmeijer et al. (2021) proposed the confidence interval method (CIM) for conducting inference on β. CIM uses working confidence intervals of β to cluster instruments and picks the largest cluster of instruments with overlapping confidence intervals. Specifically, for each instrument j, CIM first constructs p working confidence interval ${\text{CI}}_{j}\left(q_{n}\right)$ of β:

$${\text{CI}}_{j}\left(q_{n}\right)=\left[{\overset{ˆ}{\beta }}_{j}-q_{n}\cdot \hat{\text{se}}\left({\overset{ˆ}{\beta }}_{j}\right),{\overset{ˆ}{\beta }}_{j}+q_{n}\cdot \hat{\text{se}}\left({\overset{ˆ}{\beta }}_{j}\right)\right],\;{\tilde{\beta }}_{j}=\frac{{\hat{\Gamma }}_{j}}{{\overset{ˆ}{\gamma }}_{j}}.$$

The term $\hat{\text{se}}\left({\overset{ˆ}{\beta }}_{j}\right)$ is the standard error of ${\tilde{\beta }}_{j}$ based on applying the delta method to Equation 8. The parameter $q_{n}$, which depends on the sample size n, is set to measure the similarity between confidence intervals. Note that $q_{n}$ is not equal to $z_{1-\alpha /2}$, the 1-α/2 quantile of the standard normal distribution. Instead, Windmeijer et al. (2021) proposed an adaptive approach to set $q_{n}$ based on a downward testing procedure of Andrews (1999). Second, the procedure constructs K≤p subgroups of instruments where all instruments in the subgroup have overlapping working confidence intervals:

$$V_{k}=\left\{j|{\text{CI}}_{j}(q)\cap {\text{CI}}_{j^{\prime }}(q)\neq \emptyset \forall j,j^{\prime }\in \{1,\ldots ,p\}\right\},\;k=1,\ldots ,K.$$

The CIM estimator of 𝒱 is the largest subset of instruments where all the confidence intervals in the subset overlap with each other:

$${\overset{ˆ}{𝒱}}_{\text{CIM}}=\left\{V_{k}|\left|V_{k}\right|=\underset{k^{\prime }=1,\ldots ,K}{\text{max}}\left|V_{k^{\prime }}\right|\right\}.$$

Windmeijer et al. (2021) showed that if we construct a TSLS estimator of β with ${\overset{ˆ}{𝒱}}_{\text{CIM}}$, the estimator, denoted as ${\overset{ˆ}{\beta }}_{\text{CIM}}$, is consistent, asymptotically normal, and oracle-optimal.

While both TSHT and CIM produce confidence intervals for β and are oracle-optimal, a major downside of both procedures is that they rely on correctly estimating the set of valid instruments asymptotically, i.e., ${\text{lim}}_{n\to \infty }P(\overset{ˆ}{𝒱}=𝒱)=1$; this property is sometimes referred to as selection consistency. As noted in the postselection inference literature (e.g., Leeb & Pötscher 2005, Berk et al. 2013), relying on selection consistency to enable inference on β can lead to poor finite-sample properties, such as inflated type I errors. This phenomenon is exacerbated when the true π is close to 0 so that selection consistency is not guaranteed. The next section highlights some progress in this area by constructing uniformly valid confidence intervals of β.

#### 2.3.3. Uniformly valid inference of β.

There are two main procedures that construct uniformly valid confidence intervals of β. Kang et al. (2022) proposed to take a union of several confidence intervals of β constructed from subsets of instruments that pass the J test (Hansen 1982). Specifically, suppose the investigator believes that at least v≥1 instruments are valid (i.e., |𝒱|≥v) and wants to construct a 1-α confidence interval of β. The union confidence interval, denoted as ${\text{CI}}_{\text{union}}$, takes a union of $1-\alpha_{t}$ confidence intervals of β that use v instruments and the J test does not reject the null with the v instruments at level $\alpha_{s}$:

$${\text{CI}}_{\text{union}}^{\left[v\right]}=\cup_{{𝒱}^{\prime },\left|{𝒱}^{\prime }\right|=v}\left\{{\text{CI}}_{1-\alpha_{t}}\left({𝒱}^{\prime }\right)|J\left({𝒱}^{\prime }\right)\leq q_{1-\alpha_{s}}\right\},\;\alpha =\alpha_{s}+\alpha_{t},\;|𝒱|\geq v\geq 1.$$

Here, ${\text{CI}}_{1-\alpha_{t}}\left({𝒱}^{\prime }\right)$ is the $1-\alpha_{t}$ confidence interval of β using ${𝒱}^{\prime }$ as valid instruments, $J\left({𝒱}^{\prime }\right)$ is the J test using ${𝒱}^{\prime }$ as valid instruments, and $q_{1-\alpha_{s}}$ is the $1-\alpha_{s}$ quantile of the J test under its null hypothesis. The confidence interval ${\text{CI}}_{1-\alpha_{t}}$ can be any confidence interval of β that has the desired $1-\alpha_{t}$ coverage if valid instruments are used. For example, ${\text{CI}}_{1-\alpha_{t}}$ can be the Wald confidence interval from the TSLS estimator in Equation 7, the Anderson–Rubin confidence interval (Anderson & Rubin 1949), or the confidence interval based on inverting the conditional likelihood ratio test (Moreira 2003). Also, the terms $\alpha_{s}$ and $\alpha_{t}$ satisfy the constraint $\alpha_{s}+\alpha_{t}=\alpha$. For instance, choosing $\alpha_{s}=0.01$ and $\alpha_{t}=0.04$ would lead to a 95% confidence interval for ${\text{CI}}_{\text{union}}^{\left[v\right]}$. A main advantage of the union confidence interval is that it does not rely on selection consistency and is guaranteed to yield a 1-α confidence interval of β. However, the procedure requires an exponential number of computations and is generally infeasible for a moderate to a large number of instruments.

Guo (2023) proposed another approach to construct a uniformly valid confidence interval of β. To illustrate the main idea, we focus on the case where the majority rule holds (for the case under the plurality rule, see Guo 2023). Guo (2023) proposed the searching confidence interval of β based on the relationship between Γ and γ in Equation 4:

13. $${\text{CI}}_{\text{search}}=\left\{\beta \in R:L(\beta )>\frac{p}{2}\right\},\;L\left(\beta \right)=\sum_{j=1}^{p}I\left[\left|{\hat{\Gamma }}_{j}-\beta {\overset{ˆ}{\gamma }}_{j}\right|\leq z_{1-\frac{\alpha }{2p}}\cdot \hat{\text{se}}\left({\hat{\Gamma }}_{j}-\beta {\overset{ˆ}{\gamma }}_{j}\right)\right].$$

The term $\hat{\text{se}}\left({\hat{\Gamma }}_{j}-\beta {\overset{ˆ}{\gamma }}_{j}\right)$ is the estimated standard error of ${\hat{\Gamma }}_{j}-\beta {\overset{ˆ}{\gamma }}_{j}$ using the delta method, and I[⋅] is the indicator function. The term L(β) measures the number of valid instruments for a particular value of β, and the interval ${\text{CI}}_{\text{search}}$ collects all β values that lead to more than 50% of instruments being valid. Also, to reduce the length of ${\text{CI}}_{\text{search}}$, Guo (2023) proposed a sampling version of ${\text{CI}}_{\text{search}}$, denoted as ${\text{CI}}_{\text{ss}}$. The sampling confidence interval starts by resampling $\left(\overset{ˆ}{\Gamma },\overset{ˆ}{\gamma }\right)$ a number of times M based on Equation 8:

Γˆ[m]γˆ[m]~iidNΓˆγˆ,1nΩˆΓΩˆΓγΩˆΓγ⊤Ωˆγ,1≤m≤M,

where the terms ${\overset{ˆ}{\Omega }}_{\Gamma },{\overset{ˆ}{\Omega }}_{\Gamma \gamma }$, and ${\overset{ˆ}{\Omega }}_{\gamma }$ denote consistent estimators of $\Omega_{\Gamma },\Omega_{\Gamma \gamma }$, and $\Omega_{\gamma }$, respectively. We then recompute the searching confidence interval in Equation 13 from the resampled (${\overset{ˆ}{\Gamma }}^{\left[m\right]},{\overset{ˆ}{\gamma }}^{\left[m\right]}$):

14. $${\text{CI}}^{\left[m\right]}=\left\{\beta \in R:L^{\left[m\right]}(\beta )>\frac{p}{2}\right\},L^{\left[m\right]}(\beta )=\sum_{j=1}^{p}I\left[\left|{\hat{\Gamma }}_{j}^{\left[m\right]}-\beta {\overset{ˆ}{\gamma }}_{j}^{\left[m\right]}\right|\leq \lambda \cdot z_{1-\frac{\alpha }{2p}}\cdot \hat{\text{se}}\left({\hat{\Gamma }}_{j}-\beta {\overset{ˆ}{\gamma }}_{j}\right)\right].$$

The main difference between Equations 13 and 14 is the shrinkage parameter λ in Equation 14 with $\lambda =c_{n}{\left(M^{-1}\;\text{log}\;n\right)}^{\frac{1}{2p}}$ where $c_{n}$ is a data-dependent parameter (for details, see Guo 2023). The sampling confidence interval aggregates nonempty intervals of ${\text{CI}}^{\left[m\right]}$ by taking the lower and the upper limit of the interval ${\text{CI}}^{\left[m\right]}$, denoted as $l^{\left[m\right]}$ and $u^{\left[m\right]}$, respectively:

$${\text{CI}}_{\text{ss}}=\left(\underset{m\in ℳ}{\text{min}}l^{\left[m\right]},\underset{m\in ℳ}{\text{max}}u^{\left[m\right]}\right),\;ℳ=\left\{1\leq m\leq M:{\text{CI}}_{\text{search}}^{\left[m\right]}\neq \emptyset \right\}.$$

Guo (2023) showed that both ${\text{CI}}_{\text{search}}$ and ${\text{CI}}_{\text{ss}}$ achieve the desired coverage level even in the presence of instrumental variable selection error. Also, the lengths of both intervals are of the order $1/\sqrt{n}$. In finite samples, we observe that ${\text{CI}}_{\text{search}}$ and ${\text{CI}}_{\text{ss}}$ are longer than the confidence intervals from TSHT and CIM, which is to be expected since ${\text{CI}}_{\text{search}}$ and ${\text{CI}}_{\text{ss}}$ guarantee uniform coverage of β.

### 2.4. Connection to Two-Sample Summary Data Design in Mendelian Randomization

Linear models often serve as the data-generating model for a popular study design in MR called the two-sample summary data design (Pierce & Burgess 2013, Burgess et al. 2013). In this section, we briefly discuss the connection between the assumptions underlying two-sample summary data designs in MR and the assumptions discussed in the prior sections. For a full review of assumptions underlying MR, readers are directed to Bowden et al. (2017), Slob & Burgess (2020), and Sanderson et al. (2022).

Briefly, two-sample summary data designs assume that the data are generated from two independent samples and only summary statistics, usually estimates of $\overset{ˆ}{\Gamma }$ and $\overset{ˆ}{\gamma }$ along with their corresponding standard errors, are available. The summary statistics are assumed to follow a multivariate normal distribution with diagonal covariance matrices. These assumptions are formalized as follows:

15. $$\Gamma =\beta \gamma +\pi ,\;{\hat{\Gamma }}_{j}\overset{\text{ind}}{\sim }N\left(\Gamma_{j},\sigma_{j}^{2}\right),\;{\overset{ˆ}{\gamma }}_{j}\overset{\text{ind}}{\sim }N\left(\gamma_{j},\omega_{j}^{2}\right),\;\text{and}\;\overset{ˆ}{\Gamma }⊥\overset{ˆ}{\gamma }.$$

Additional details are provided by Bowden et al. (2017), Zhao et al. (2020), and Ye et al. (2021). In terms of identification, two-sample summary data designs assume the same relationship Γ=βγ+π in Equation 4. Also, many methods in two-sample summary data MR make similar assumptions about π to those in Section 2.2. For example, Bowden et al. (2016) proposed the weighted median estimator of β, which is consistent whenever the majority rule in Equation 5 holds. Hartwig et al. (2017) proposed the zero modal pleiotropy assumption, which is the MR version of the plurality rule in Equation 6, and showed that their proposed modal estimator of β is consistent. An improvement of the modal estimator was proposed by Burgess et al. (2018). Yao et al. (2023) proposed MR by selecting valid instruments and then performing post-selection inference. This method is a modified version of the methods proposed by Guo et al. (2018) and Guo (2023). Bowden et al. (2015) proposed the instrument strength independent of direct effect (InSIDE) assumption, which is the MR version of the orthogonality condition in Equation 11. We remark that the InSIDE assumption is similar to balanced horizontal pleiotropy (Bowden et al. 2017, Hemani et al. 2018, Zhao et al. 2020), which states that $\pi_{j}\overset{\text{ind}}{\sim }N\left(0,\tau^{2}\right)$ and $\pi_{j}s$ are independent of $\overset{ˆ}{\Gamma }$ and $\overset{ˆ}{\gamma }$.

In terms of inference, two-sample summary data designs in Equation 15 place stronger assumptions on $\overset{ˆ}{\Gamma }$ and $\overset{ˆ}{\gamma }$ than the one-sample individual data designs in prior sections. Specifically, Equation 15 assumes that $\overset{ˆ}{\Gamma }$ and $\overset{ˆ}{\gamma }$ are exactly normal and entries of the vector $\left(\overset{ˆ}{\Gamma },\overset{ˆ}{\gamma }\right)$ are independent of each other. This is stronger than Equation 8, where there can be dependence among $\left(\overset{ˆ}{\Gamma },\overset{ˆ}{\gamma }\right)$, and $\left(\overset{ˆ}{\Gamma },\overset{ˆ}{\gamma }\right)$ only have to be asymptotically normal.

## 3. NONLINEAR MODELS

Recent lines of research have utilized nonlinearities in the exposure model to identify and estimate β in the presence of invalid instruments. The main idea is to leverage nonlinear trends in the exposure model to create new instruments, which are then used to identify and estimate the causal effect of the exposure. Compared with linear methods, nonlinear treatment methods enable causal identification even when the plurality or majority assumptions are violated. But, as mentioned in Section 1, investigators should verify that the exposure model is indeed nonlinear to ensure that these methods yield valid results. In this section, we review two recent methods in this area.

First, Guo et al. (2022) considered the following modifications of Equation 1:

$$\begin{array}{cccc} D=f(Z)+\delta , & \; & \text{E}(\delta |Z)=0,\text{Var}\left[{𝒫}_{Z}^{⊥}f(Z)\right]>0, & \text{E}[f(Z)]=0,\text{E}(Z)=0. \end{array}$$

The term Z is a p-dimensional random variable, and the term ${𝒫}_{Z}^{⊥}f(Z)=f(Z)-Z\tilde{\gamma },\tilde{\gamma }={\text{argmin}}_{\gamma }\text{E}[f(Z)-Z\gamma {]}^{2}$, is the residual from the best linear approximation of f(Z). The positive variance assumption $\text{Var}\left[{𝒫}_{Z}^{⊥}f(Z)\right]>0$ ensures that f(Z) is a nonlinear function of Z. More generally, the positive variance assumption states that the exposure D can be explained through linear and nonlinear functions of Z. In contrast, the outcome’s relationship with the instruments in Equation 2 is linear. Critically, this discrepancy allows opportunities to create a nonlinear instrument and identify β with invalid instruments. To put it differently, the nonlinearity assumption on f guarantees that the association between the treatment and the instrument, which is also characterized by the function f(⋅), is more complicated than the functional form of the violation, which is linear. We can formalize this observation by noticing that at the true value of β, the following equation holds:

$$\text{E}\left\{\left[{𝒫}_{Z}^{⊥}f(Z)\right]\cdot (Y-D\beta )\right\}=\text{E}\left\{\left[{𝒫}_{Z}^{⊥}f(Z)\right]\cdot \left(Z^{⊤}\pi \right)\right\}=0.$$

The first equality follows from E[ϵ∣Z]=0 in Equation 2, and the second equality follows from the property of the orthogonal projection ${𝒫}_{Z}^{⊥}$. Guo et al. (2022) also discuss a generalization of the above observation to the case where the instruments’ direct effect on the outcome is nonlinear.

For estimation, Guo et al. (2022) proposed the following two-step method with sample splitting. Suppose we randomly split the data into two parts, where the data in the first part are indexed as $i=1,\ldots ,n_{1}$ and the data in the second part are indexed as $i=n_{1}+1,\ldots ,n$. In the first step, f(Z) is estimated using a nonparametric estimator or a machine learning algorithm (e.g., random forests) based on the data from the second part. In the second step, the fitted function f is evaluated in the data from the first part, and Guo et al. (2022) showed that this fit can be represented as

$$\left[\begin{array}{c} \overset{ˆ}{f}\left(Z_{1}\right)\\ \vdots \\ \overset{ˆ}{f}\left(Z_{n_{1}}\right) \end{array}\right]=Q\tilde{D},\;\tilde{D}=\left(\begin{array}{c} D_{1}\\ \vdots \\ D_{n_{1}} \end{array}\right)\in R^{n_{1}\times 1},\;Q\in R^{n_{1}\times n_{1}}.$$

The matrix Q can be thought of as a matrix representation of the nonparametric estimator used in the second part of the data. For example, if f(Z) is estimated via split random forests, each row of the matrix Q represents a $n_{1}$-dimensional aggregation weight (Lin & Jeon 2006, Meinshausen 2006, Wager & Athey 2018). Let

$$\tilde{Y}=Q\left(\begin{array}{c} Y_{1}\\ \vdots \\ Y_{n_{1}} \end{array}\right)\in R^{n_{1}\times 1},\;\tilde{Z}=Q\left(\begin{array}{c} Z_{1}\\ \vdots \\ Z_{n_{1}} \end{array}\right)\in R^{n_{1}\times p},\;M=Q^{⊤}P_{\tilde{Z}}^{⊥}Q\in R^{n_{1}\times n_{1}}.$$

Then, we introduce the following bias-corrected estimator of β,

16. $${\overset{ˆ}{\beta }}_{\text{TSCI}}=\tilde{\beta }-\frac{\sum_{i=1}^{n_{1}}M_{i,i}{\overset{ˆ}{\delta }}_{i}{\overset{ˆ}{\epsilon }}_{i}}{{\tilde{D}}^{⊤}M\tilde{D}},\;\tilde{\beta }=\frac{Y^{⊤}MD}{D^{⊤}MD},$$

where ${\overset{ˆ}{\delta }}_{i}=D_{i}-\overset{ˆ}{f}\left(Z_{i}\right)$ and ${\overset{ˆ}{\epsilon }}_{i}$ is the ith element of the vector $P_{Z}^{⊥}(Y-D\tilde{\beta })$. Because identification of β relies on the nonlinear curvature ${𝒫}_{Z}^{⊥}f(Z)$ and the estimation of β uses a two-step procedure, the estimator ${\overset{ˆ}{\beta }}_{\text{TSCI}}$ is referred to as the two-stage curvature identification (TSCI) estimator.

Second, Sun et al. (2023) proposed to use higher-order interactions of p instruments in the exposure model to identify β. Similar to Guo et al. (2022), Sun et al. (2023) generated new instruments from the p instruments, and the new instruments have nonlinear effects on the exposure. However, Guo et al. (2022) used nonparametric estimators to create these new instruments, whereas Sun et al. (2023) used higher-order interactions to create them. A bit more formally, suppose all p instruments are mutually independent and there are at least v valid instruments (i.e., |𝒱|≥v and v≥1) [when the instruments are dependent, see Sun et al. (2023)]. Then, using the G-estimation framework (Robins et al. 1992, Robins 1994), Sun et al. (2023) showed that there is a function $h^{\left[v\right]}(Z)\in R^{d}$ with d=∑j=0v-1pj such that β is the unique solution to

17. $$\text{E}\left[h^{\left[v\right]}(Z)(Y-\beta D)\right]=0,\;\left|𝒱\right|\geq v,$$

if $h^{\left[v\right]}(Z)$ is associated with the exposure D. The function $h^{\left[v\right]}(Z)$ represents all higher-order interactions of p instruments of order greater than or equal to v. Specifically, for each j=0,…,v-1, we create all possible subsets of p instruments of size p-j and denote this set as ${𝒞}_{j}$:

$${𝒞}_{0}=\left\{\left(1,\ldots ,p\right)\right\},$$

$${𝒞}_{1}=\{(1,\ldots ,p-1),(1,\ldots ,p-2,p),\ldots ,(2,\ldots ,p)\},$$

$${𝒞}_{j}=\left\{C\subseteq \{1,\ldots ,p\}|\left|C\right|=p-j\right\},\;j=0,\ldots ,v-1.$$

Then, for each element $C\in {𝒞}_{j}$, we create the interaction instrument $\Pi_{k\in C}\left[Z_{j}-\text{E}\left(Z_{j}\right)\right]$. For example, for ${𝒞}_{0}$ and ${𝒞}_{1}$, we have the following interaction instruments:

$${𝒞}_{0}\Rightarrow \prod_{k=1}^{p}\left[Z_{k}-\text{E}\left(Z_{k}\right)\right],$$

$${𝒞}_{1}\Rightarrow \prod_{k\neq p}\left[Z_{k}-\text{E}\left(Z_{k}\right)\right],\prod_{k\neq p-1}\left[Z_{k}-\text{E}\left(Z_{k}\right)\right],\ldots ,\prod_{k\neq 1}\left[Z_{k}-\text{E}\left(Z_{k}\right)\right].$$

Stacking all the interaction instruments generated by every ${𝒞}_{j},j=0,\ldots ,v-1$ into a vector defines the function $h^{\left[v\right]}(Z)$. Or, in other words, $h^{\left[v\right]}(Z)$ creates d interaction instruments.

As an illustrative example, suppose we have p=2 instruments $Z=\left(Z,Z^{\prime }\right),Z$ and $Z^{\prime }$ are mutually independent, and at least one of them is valid (i.e., v=1). Then, d=∑j=0v-1pj=1,𝒞0={{1,2}}, and $h^{\left[1\right]}(Z)=[Z-\text{E}(Z)]\left[Z^{\prime }-\text{E}\left(Z^{\prime }\right)\right]$. As long as $[Z-\text{E}(Z)]\left[Z^{\prime }-\text{E}\left(Z^{\prime }\right)\right]$ is associated with D,β is the unique solution of Equation 17 because

$$\text{E}\left[\left(Z-\text{E}[Z])\left(Z^{\prime }-\text{E}\left[Z^{\prime }\right]\right)(Y-\beta D\right)\right]=\text{E}\left[(Z-\text{E}[Z])\left(Z^{\prime }-\text{E}\left[Z^{\prime }\right]\right)Z_{{𝒱}^{C}}\pi_{{𝒱}^{C}}\right]=0.$$

From the law of total expectations, the last equality holds for any 𝒱 so long as |𝒱|≥v=1 and the two instruments are independent of each other. Also, the last equality continues to hold for any 𝒱 with |𝒱|≥1 even if the term $Z_{{𝒱}^{C}}\pi_{{𝒱}^{c}}$ in Equation 2 is nonlinear, for instance if $Z_{{𝒱}^{C}}\pi_{{𝒱}^{c}}$ is replaced by an unknown function $\pi \left(Z_{{𝒱}^{c}}\right)$. Or, more loosely stated, in addition to Z and $Z^{\prime },h^{\left[1\right]}(Z)$ serves as the new interaction instrument, and so long as one of these instruments is valid, we can still identify the parameters in Equation 2.

For estimation, we can replace the terms in Equation 17 with their empirical counterparts. Or, we can run TSLS with the interaction instruments $h^{\left[v\right]}(Z)$, and the term $\text{E}\left(Z_{j}\right)$ in $h^{\left[v\right]}(Z)$ is replaced by the jth column mean of the instrument matrix $Z\in R^{n\times p}$. In the example above where we have two instruments and v=1, the estimator simplifies to

$${\overset{ˆ}{\beta }}_{\text{g}}^{\left[1\right]}=\frac{\sum_{i=1}^{n}\left(Z_{i}-\overset{‾}{Z}\right)\left(Z_{i}^{\prime }-{\overset{‾}{Z}}^{\prime }\right)Y_{i}}{\sum_{i=1}^{n}\left(Z_{i}-\overset{‾}{Z}\right)\left(Z_{i}^{\prime }-{\overset{‾}{Z}}^{\prime }\right)D_{i}}.$$

The terms $\overset{‾}{Z}$ and ${\overset{‾}{Z}}^{\prime }$ represent the means of Z and $Z^{\prime }$, respectively. The statistical properties of ${\overset{ˆ}{\beta }}_{\text{g}}^{\left[v\right]}$ can be established by using the M-estimation framework. Sun et al. (2023) also proposed a new multiply robust identification framework and a semiparametric efficient estimator of β.

## 4. HETEROSKEDASTIC MODELS

Another approach to studying the causal effect of the exposure with invalid instruments is by leveraging heteroskedasticity of the observed data (Lewbel 2012, Tchetgen Tchetgen et al. 2021, Sun et al. 2022, Ye et al. 2024). Specifically, consider the following variations of Equations 1 and 2:

18. $$\text{E}\left(Y|D,Z,U\right)=\beta D+Z\pi +\xi_{y}\left(U\right),$$

19. $$\begin{array}{c} \text{E}\left(D|Z,U\right)=Z\gamma +\xi_{d}\left(U\right),\;Z⊥U, \end{array}$$

where $\xi_{y},\xi_{d}$ are unspecified functions. The variable U represents an unmeasured variable that affects both the outcome Y and the exposure D. Tchetgen Tchetgen et al. (2021) showed that under Equations 18 and 19, β can be identified as the unique solution to the estimating equation

20. E{[Z-E(Z)][D-E(D∣Z)](Y-βD)}=0

as long as D is heteroskedastic, i.e., Var(D∣Z) varies as a function of Z. Specifically, under Equations 18 and 19, the left-hand side of Equation 20 simplifies to

$$\text{E}\{[Z-\text{E}(Z)][D-\text{E}(D|Z)][\text{E}(Y|D,Z,U)-\beta D]\}\;=\text{E}\left\{[Z-\text{E}(Z)][D-\text{E}(D|Z)]\left[Z\pi +\xi_{y}(U)\right]\right\}\;=\text{E}\left\{\left[Z-\text{E}(Z)][D-\text{E}(D|Z)]\xi_{y}(U\right)\right\}\;=\text{E}\left\{\left[Z-\text{E}(Z)]\left[\xi_{d}(U)-\text{E}\left[\xi_{d}(U)\right]\right]\xi_{y}(U\right)\right\}\;=\text{E}[Z-\text{E}(Z)]\text{E}\left\{\left[\xi_{d}(U)-\text{E}\left[\xi_{d}(U)\right]\right]\xi_{y}(U)\right\}\;=0.$$

The above argument holds more broadly even if we replace Zπ in Equation 18 with any function Z or if we replace Zγ in Equation 19 with any function of Z. Tchetgen Tchetgen et al. (2021) provide more general conditions under which Equation 20 holds and refer to this framework as G-estimation under no interaction with unmeasured selection (GENIUS).

Intuitively, Equation 20 constructs p new interaction instruments of the form [Z-E(Z)][D-E(D∣Z)], which are the product of the original candidate set of instruments Z and the residual D-E(D∣Z). From the independence of U and Z, the residual D-E(D∣Z) is a proxy for $\xi_{d}(U)$. Then, the interaction instruments are valid instruments in that since there are no interactions between U and Z in Equation 18, the interaction instruments satisfy the no direct effect assumption in condition b. Also, the interaction instruments are correlated with the exposure where, for any j=1,…,p, we have

$$\text{Cov}\left\{\left[Z_{j}-\text{E}\left(Z_{j}\right)\right][D-\text{E}(D|Z)],D\right\}\;=\text{E}\left\{\left[Z_{j}-\text{E}\left(Z_{j}\right)\right][D-\text{E}(D|Z)]D\right\}\;=\text{E}\left\{\left[Z_{j}-\text{E}\left(Z_{j}\right)\right]\text{Var}(D|Z)\right\}+\text{E}\left\{\left[Z_{j}-\text{E}\left(Z_{j}\right)\right][D-\text{E}(D|Z)]\text{E}(D|Z)\right\}\;=\text{E}\left\{\left[Z_{j}-\text{E}\left(Z_{j}\right)\right]\text{Var}(D|Z)\right\}\neq 0.$$

The first equality uses the definition of covariance, and the second equality uses D=D-E[D∣Z]+E[D∣Z] along with the definition of conditional variance. The third equality uses the law of total expectation that conditions on Z, and the final inequality is due to heteroskedasticity of D. Combined, the interaction instruments [Z-E(Z)][D-E(D∣Z)] are new instruments to identify β in Equation 18. Note that this approach is similar to the one in Section 3 where higher-order interactions of Z are used to create new instruments and identify β. Also, because the above identification strategy relies on heteroskedasticity of the exposure D to create the new interaction instruments, it is possible to identify β in Equation 18 even if all instruments have direct effects on the outcome, i.e., if $\pi_{j}\neq 0$ for all j.

For estimation, one simple approach is to solve the sample equivalent version of Equation 20:

$${\overset{ˆ}{\beta }}_{\text{GENIUS}}=\underset{\beta }{\text{argmin}}\sum_{i=1}^{n}{\left[\left(Z_{i}-\bar{Z}\right)\left(D_{i}-Z_{i}\overset{ˆ}{\gamma }\right)\left(Y_{i}-\beta D_{i}\right)\right]}^{⊤}\left[\left(Z_{i}-\bar{Z}\right)\left(D_{i}-Z_{i}\overset{ˆ}{\gamma }\right)\left(Y_{i}-\beta D_{i}\right)\right].$$

The estimator ${\overset{ˆ}{\beta }}_{\text{GENIUS}}$ replaces E[D∣Z] with an estimate from a linear regression model where we regress D on Z. Under some moment assumptions, Tchetgen Tchetgen et al. (2021) show that ${\overset{ˆ}{\beta }}_{\text{GENIUS}}$ is consistent and asymptotically normal for β. Ye et al. (2024) present an extension of this estimator that is robust to many weak invalid instruments, and Sun et al. (2022) propose a multiply robust estimator of β where they use machine learning estimators for estimating relevant nuisance functions (e.g., E[D∣Z]).

Finally, we discuss another idea based on heteroskedasticity, proposed by Liu et al. (2023). Following previous notations, the authors considered the following variation of Equation 2:

21. $$Y=\beta_{0}+\beta D+Z\pi +\alpha D\;\text{exp}\left(\eta_{0}+Z\eta \right)+\xi ,\;\xi |D,Z\sim N\left[0,\text{exp}\left(\eta_{0}+Z\eta \right)\right],\eta \neq 0.$$

Here, the parameter β represents the average treatment effect on the treated and is the target parameter of interest. Equation 21 is the observable implication of the following identification assumptions: (a) no additive interaction between D and Z (i.e., E[Y(d,z)-Y(0,z)∣D=d,Z=z]=dβ for all z), (b) homogeneous confounding of D, and (c) the outcome Y among D=0 following a normal distribution with variance $\text{exp}\left(\eta_{0}+Z\eta \right)$ and η≠0. Condition a is weaker than the constant effect assumption, where Y(d,z)-Y(0,z)=dβ, and is satisfied if Z does not modify the average treatment effect on the treated. Condition b is defined on the odds ratio scale and encodes the assumption that confounding on D does not depend on Z (for additional discussion, see Liu et al. 2023). Conditions b and c give rise to the term $\alpha D\;\text{exp}\left(\eta_{0}+Z\eta \right)+\xi$ in Equation 21. The distributional assumption can be relaxed to a mixture of normal distributions or to a distribution of Y given D=0 that is heteroskedastic in Z. Finally, estimation of β is based on the likelihood principle, where we maximize the log likelihood of $\text{P}\left(Y_{i}|D_{i},Z_{i}\right)$ in Equation 21; we denote this estimator ${\overset{ˆ}{\beta }}_{\text{MiSTERI}}$, where MiSTERI stands for mixed-scale treatment effect robust identification. Another estimation approach based on the method of moments is discussed by Liu et al. (2023).

## 5. ILLUSTRATION WITH REAL DATA

### 5.1. Background and Setup

We demonstrate the methods introduced above by reanalyzing the effect of BMI on systolic blood pressure (SBP) from the UK Biobank (for details, see Sun et al. 2023, section 5). Briefly, the UK Biobank is a large-scale prospective cohort study that recruited roughly 500,000 participants between 2006 and 2010 in the United Kingdom (Sudlow et al. 2015). In the dataset, BMI was measured in units of kilograms per meter squared and SBP was measured in units of millimeters of mercury. Following Sun et al. (2023), we restrict our analysis to people of genetically verified white British descent (Tyrrell et al. 2016) and who are not taking antihypertensive medication based on self-reporting. The sample size for the final analysis is n=292,757. We use the top p=10 single nucleotide polymorphisms (SNPs) ranked by their p-values, each of which were derived from testing the effect of a SNP on BMI with simple linear regression. The 10 p-values reach a genome-wide significance level of 5 × 10^−8^ (Locke et al. 2015) and have pairwise correlation coefficients that are less than 0.01. The 10 SNPs are rs1558902, rs6567160, rs543874, rs13021737, rs10182181, rs2207139, rs11030104, rs10938397, rs13107325, and rs3888190. The overall F-statistic for the first-stage model (i.e., Equation 1 with 10 instruments) is 146.1, with a p-value that is less than 10^−8^. To focus on problems caused by invalid instruments, we choose the top 10 SNPs here to minimize effects from weak instruments; see Sun et al. (2023) for further details on how these instruments were chosen.

We compare the following methods for estimating $\beta :{\overset{ˆ}{\beta }}_{\text{med}},{\overset{ˆ}{\beta }}_{\text{sisVIVE}},{\overset{ˆ}{\beta }}_{\text{kclass}}\;{\overset{ˆ}{\beta }}_{\text{AdLasso}},{\overset{ˆ}{\beta }}_{\text{TSHT}},{\overset{ˆ}{\beta }}_{\text{CIM}},{\overset{ˆ}{\beta }}_{\text{TSCI}},{\overset{ˆ}{\beta }}_{\text{g}}^{\left[v\right]},{\overset{ˆ}{\beta }}_{\text{GENIUS}}$, and ${\overset{ˆ}{\beta }}_{\text{MiSTERI}}$. For ${\overset{ˆ}{\beta }}_{\text{g}}^{\left[v\right]}$, we set the minimum number of valid instruments to be 6 and 8, and they are denoted as ${\overset{ˆ}{\beta }}_{\text{g}}^{\left[6\right]}$ and ${\overset{ˆ}{\beta }}_{\text{g}}^{\left[8\right]}$, respectively. We also compute the union confidence interval ${\text{CI}}_{\text{union}}^{\left[v\right]}$ and the search and sampling confidence interval ${\text{CI}}_{\text{ss}}$. For ${\text{CI}}_{\text{union}}^{\left[v\right]}$, we set the minimum number of valid instruments to be v=6 and v=8 and use the conditional likelihood ratio test (Moreira 2003). Finally, we include two baseline analyses of the causal effect. The first baseline analysis is the ordinary least squares estimator of β that fits a linear regression of SBP on BMI. The second baseline analysis is the TSLS estimator of β from Section 2.3.1 that sets 𝒱 to be all 10 SNPs; in other words, this estimator assumes that all 10 SNPs are valid.

We use the following software to compute the estimates or confidence intervals of β. To compute ${\overset{ˆ}{\beta }}_{\text{sisVIVE}}$, we use the R package sisVIVE (Kang 2017). To compute ${\overset{ˆ}{\beta }}_{\text{kclass}}$, we use the R package ivmodel (Kang et al. 2021). To compute ${\overset{ˆ}{\beta }}_{\text{med}}$ and ${\overset{ˆ}{\beta }}_{\text{AdLasso}}$, we use the code provided by Windmeijer et al. (2019). To compute ${\overset{ˆ}{\beta }}_{\text{TSHT}}$ and the search and sampling confidence interval ${\text{CI}}_{\text{ss}}$, we use the R package RobustIV (Koo et al. 2023). To compute the union confidence interval ${\text{CI}}_{\text{union}}^{\left[v\right]}$, we use the code provided by Kang et al. (2022). To compute ${\overset{ˆ}{\beta }}_{\text{CIM}}$, we use the code provided by Windmeijer et al. (2021). To compute ${\overset{ˆ}{\beta }}_{\text{TSCI}}$, we use R package TSCI (Carl et al. 2023). To compute ${\overset{ˆ}{\beta }}_{\text{g}}^{\left[v\right]}$, we use the code in the R package MRSquare. Finally, to compute ${\overset{ˆ}{\beta }}_{\text{GENIUS}}$ and ${\overset{ˆ}{\beta }}_{\text{MiSTERI}}$, we use the code provided by Tchetgen Tchetgen et al. (2021) and Liu et al. (2023), respectively.

### 5.2. Results

**Figure 1:**
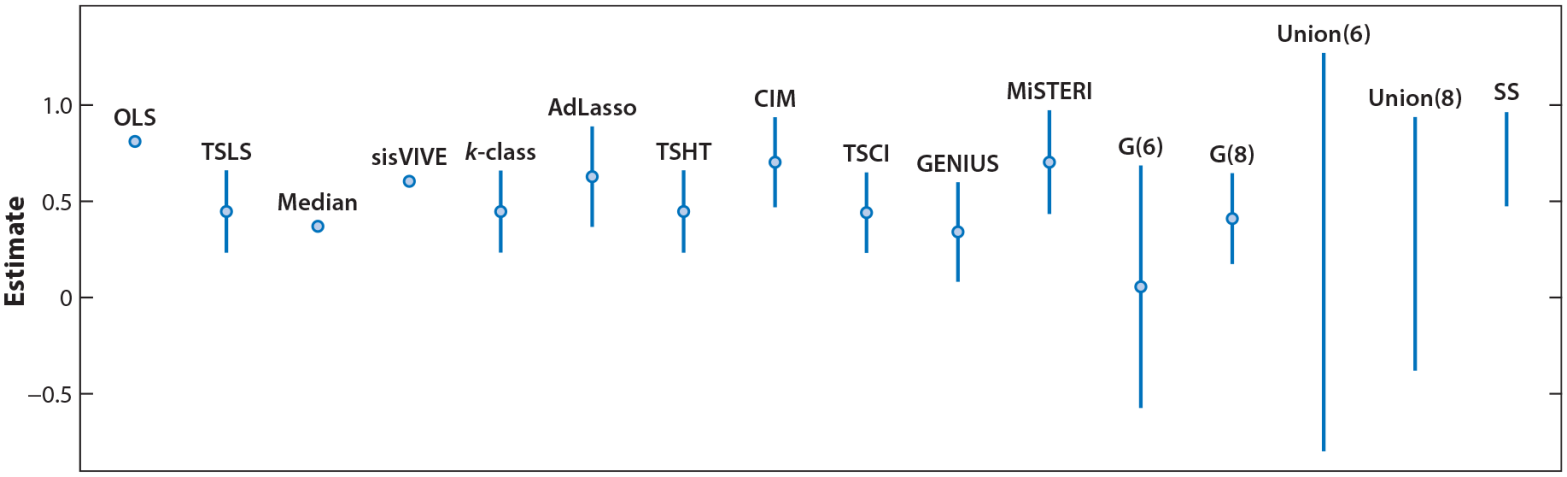
Point estimates (denoted as points) or 95% confidence intervals (denoted as an interval) for the effect of body mass index on systolic blood pressure from the UK Biobank. Sample size is n=292,757 and there are p=10 genetic instruments. OLS refers to the ordinary least squares estimator of β without using instruments. TSLS refers to ${\overset{ˆ}{\beta }}_{\text{TSLS}}$, which assumes all ten instruments are valid. Median refers to ${\overset{ˆ}{\beta }}_{\text{med}}$, sisVIVE refers to ${\overset{ˆ}{\beta }}_{\text{sisVIVE}},k$-class refers to ${\overset{ˆ}{\beta }}_{\text{kclass}}$, AdLasso refers to ${\overset{ˆ}{\beta }}_{\text{AdLasso}}$, TSHT refers to ${\overset{ˆ}{\beta }}_{\text{TSHT}}$, CIM refers to ${\overset{ˆ}{\beta }}_{\text{CIM}}$, TSCI refers to ${\overset{ˆ}{\beta }}_{\text{TSCI}}$, GENIUS refers to ${\overset{ˆ}{\beta }}_{\text{GENIUS}}$, and MiSTERI refers to ${\overset{ˆ}{\beta }}_{\text{MiSTERI}}$. G(6) and G(8) refer to ${\overset{ˆ}{\beta }}_{\text{g}}^{\left[6\right]}$ and ${\overset{ˆ}{\beta }}_{\text{g}}^{\left[8\right]}$, respectively. SS refers to ${\text{CI}}_{\text{ss}}$. Also, Union(6) and Union(8) refer to union confidence intervals that assume at least six and eight instruments are valid, respectively. Other abbreviations: AdLasso, adaptive lasso; CI, confidence interval; CIM, confidence interval method; GENIUS, G-estimation under no interaction with unmeasured selection; MiSTERI, mixed-scale treatment effect robust identification; sisVIVE, some invalid, some valid instrumental variables estimator; SS, search and sampling; TSCI, two-stage curvature identification; TSHT, two-stage hard thresholding; TSLS, two-stage least squares.

The point estimates and 95% confidence intervals for different methods are summarized in Figure 1. Except for ${\overset{ˆ}{\beta }}_{\text{g}}^{\left[6\right]},{\text{CI}}_{\text{union}}^{\left[6\right]}$, and ${\text{CI}}_{\text{union}}^{\left[8\right]}$, all methods, including the TSLS estimator that assumes all instruments are valid and the OLS estimator that does not use any instruments, suggest that there is a positive effect of increasing BMI on SBP. The largest and smallest values of the causal effect are from the union confidence interval that assumes at least 6 instruments are valid; its upper confidence limit of the causal effect is 1.2710 and its lower confidence limit is −0.799. The G-estimator that assumes at least six instruments are valid (i.e., ${\overset{ˆ}{\beta }}_{\text{g}}^{\left[6\right]}$) also gives wide confidence intervals, ranging from −0.5740 to 0.6860. But after we increase the number of valid IVs to 8, the confidence interval is (0.174, 0.646) and no longer covers 0. In general, the G-estimator and the union confidence interval allow users to conduct sensitivity analysis by varying the number of valid IVs, and can be used to reflect the uncertainty about the validity of instruments.

Among methods that generate confidence intervals, the 95% confidence intervals overlap with each other. In other words, after accounting for sampling uncertainty, these methods, despite making different assumptions about instrument invalidity, do not differ from each other with respect to their conclusions about the causal effect. Excluding the OLS estimator, the narrowest 95% confidence interval is generated from ${\overset{ˆ}{\beta }}_{\text{TSCI}}$, and the widest confidence interval is generated from the union confidence interval that assumes at least six instruments are valid.

Despite almost all methods suggesting that the effect of BMI on SBP is positive, we do see that the methods roughly cluster into two types. The first cluster of methods (i.e., sisVIVE, the adaptive lasso, CIM, MiSTERI, and the search and sampling confidence interval) roughly estimates the causal effect to be around 0.68, and they are close to the OLS estimator that does not use any instruments. The second cluster of methods (i.e., the median estimator, the k-class estimator, TSHT, TSCI, the G-estimators, GENIUS, and the union confidence intervals) roughly estimate the causal effect to be around 0.40, and they are close to the TSLS estimator that assumes all 10 instruments are valid.

Among methods that select valid instruments, CIM selected rs543874 and rs10182181 as invalid instruments. TSHT selected rs10182181 as an invalid instrument. The adaptive lasso selected rs543874, rs10182181, and rs13107325 as invalid instruments. Across all methods that can select valid instruments, instrument rs10182181 was selected as an invalid instrument.

## 6. DISCUSSION

This article provides a review of identification and inference of the causal effect of the exposure on the outcome when there are invalid instruments. We start with the linear model framework where the parameter π in Equation 2 encodes the violations of the IV assumptions. Broadly speaking, methods in this framework require that either the majority or a plurality of instruments are valid to obtain identification and inference of β. Subsequent methods have leveraged nonlinearities or heteroskedasticities in the models to identify and infer β. In our data analysis, we find that all methods yield similar conclusions about the effect of BMI on SBP.

Despite significant progress in identifying and inferring the causal effect of the exposure in the presence of invalid instruments, several challenges remain and we highlight a couple of them. First, as illustrated throughout the article, there are different ways to define a valid (or an invalid) instrument based on how the instrument deviates from assumptions b and c. Section 2.1 defines an invalid instrument through a linear deviation from the IV assumptions b and c. In contrast, Sections 3 and 4 define an invalid instrument through both linear and nonlinear deviations from the IV assumption. Because the latter sections allow for broader types of invalid instruments, they typically require additional conditions on the data-generating model for identification and inference, such as a nonlinear exposure model or a heteroskedastic exposure or outcome model. Second, while methods for uniform inference exist, there is still room for improvement, especially compared with the oracle TSLS estimator that knows which instruments are valid a priori. Third, only a handful of methods have explored how to conduct valid inference when instruments are both invalid and weakly associated with the exposure; these instruments are common in MR where the genetic variants only explain a fraction of the variance in the exposure and most of them are suspected to be pleiotropic. Guo et al. (2018) propose a thresholding procedure in TSHT to select instruments that are strongly correlated with the exposure. Lin et al. (2024) consider many weak instruments under the plurality rule, where instead of only using instruments that are strongly correlated with the exposure, they use both strong and weak instruments. Zhang et al. (2022) consider another approach to select strong instruments that also prevents the mis-selection of valid instruments. Ye et al. (2024) propose an improved version of the GENIUS estimator that allows for many weak invalid instruments discussed by Newey & Windmeijer (2009). The method of Kang et al. (2022) allows for uniformly valid inference in the presence of weak instruments defined by Staiger & Stock (1997) and invalid instruments defined in Definition 1. Fourth, how to select the optimal set of instruments for bias reduction and/or efficiency improvement when invalid instruments are present is still an open question. This problem is especially challenging when faced with many weak instruments (Liu et al. 2023, Lin et al. 2024, Ye et al. 2024).
